# Azacitidine-induced bullous pemphigoid-like localized toxic reaction

**DOI:** 10.31744/einstein_journal/2025RC0699

**Published:** 2025-02-26

**Authors:** Rodrigo Brêtas Emerich Nogueira, Nelson Hidekazu Tatsui, Laura Ramos de Almeida, Marcella Soares Pincelli, Celina Wakisaka Maruta, José Antonio Sanches, Elvira Deolinda Rodrigues Pereira Velloso, Youko Nukui

**Affiliations:** 1 Department of Hematology, Hemotherapy and Cell Therapy Faculdade de Medicina Universidade de São Paulo São Paulo SP Brazil Department of Hematology, Hemotherapy and Cell Therapy, Faculdade de Medicina, Universidade de São Paulo, São Paulo, SP, Brazil.; 2 Department of Dermatology Universidade de São Paulo São Paulo SP Brazil Department of Dermatology, Universidade de São Paulo, São Paulo, SP, Brazil.

**Keywords:** Pemphigoid, bullous, Azacitidine, Skin diseases, Myelodysplastic syndromes

## Abstract

Azacitidine is a hypomethylating agent recommended for the treatment of patients with high-risk myelodysplastic syndromes. Here, we report the case of a patient with myelodysplastic syndrome who was not eligible for allogeneic stem cell transplantation (allo-SCT) and presented with a rare and previously unreported cutaneous side effect after the use of subcutaneous azacitidine. We propose that changing the route of azacitidine administration from subcutaneous to intravenous could potentially decrease the occurrence of bullous pemphigoid-like localized toxic reactions in some patients.

## INTRODUCTION

Azacitidine is a hypomethylating agent recommended for treating high-risk myelodysplastic syndrome (MDS).^1^

Since 1991, multiple cutaneous adverse events associated with azacitidine have been reported.^2-4^ However, azacitidine discontinuation due to cutaneous reactions requires careful risk-benefit consideration^3^ since this medication has been demonstrated to increase overall survival relative to conventional care for higher-risk MDS patients.^1^

## CASE REPORT

In January 2022, a 76-year-old male patient with transfusion-dependent anemia was diagnosed with intermediate risk (IPSS 0.5 and R-IPSS 4.0) myelodysplastic syndrome with multilineage dysplasia and normal karyotype. The anemia was unresponsive to erythropoietin and cyclosporin A, and hypomethylation therapy was subsequently initiated.

The first outpatient cycle of subcutaneous azacitidine therapy, with an average dosage of 75 mg/m^2^/day for a 7-day duration was prescribed on December 12, 2022. Premedication was administered with hydroxyzine and ondansetron. The patient denied concurrent use of other medications or a history of allergic reactions. The first cycle of medication was uneventful, and a second cycle was prescribed.

**Figure 1:**
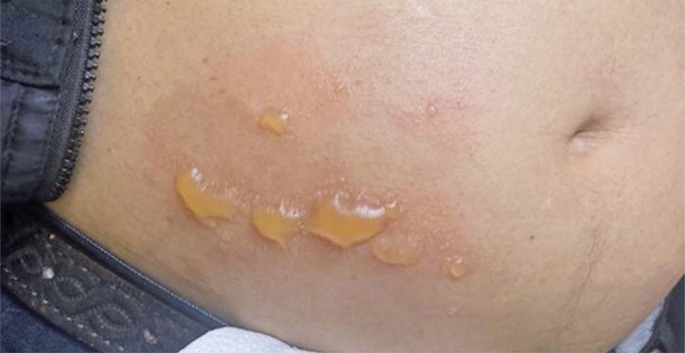
Multiple pruritic citrine bullous lesions measuring from 0.2cm to larger than 2.0cm with an erythematous base at the right iliac fossa on January 11th, 2023

The patient received the first two injections of subcutaneous azacitidine on January 9th and 10th without complications. However, on admission to the Hematology Clinic for the third dose (January 11th), the patient presented with multiple pruritic citrine bullous lesions measuring from 0.2cm to larger than 2.0cm with an erythematous base at the right iliac fossa (Figure 1), where azacitidine had been injected two days prior, with no further complaints. The patient only recalled that hours earlier he had felt a mild burning sensation and itching at his right iliac fossa just before the bullae appeared and decided to rub fine shreds of cucumber peel to his abdomen.

Since the initial hypothesis for the reaction was attributed mostly to acute toxic contact dermatitis caused by cucumber, the patient received a third dose of subcutaneous azacitidine in his hypogastric region. The next day, he presented with worsening cutaneous lesions, which also appeared on his left iliac fossa. The D4 injection was postponed and the patient was referred to the Dermatology Clinic. Hypotheses of localized bullous pemphigoid or localized toxic reactions related to the chemotherapeutic agent were formulated and the patient was prescribed topical corticosteroids.

The patient returned to the Hematology Clinic on January 17th showing residual hyperchromic lesions and dried blisters on his iliac fossae and hypogastrium, and was administered a fourth dose of azacitidine on the mesogastrium. The next day, he showed the same pattern of blistering lesions in the region, received another dose of azacitidine applied to the flanks, and underwent a skin punch biopsy. Blood tests showed normal C-reactive protein and eosinophil counts, and mildly elevated IgE and erythrocyte sedimentation rates. On January 19th and 20th, the patient received further doses of azacitidine, with small blisters continuing to appear at the injection sites, thereby completing the second cycle of the prescribed hypomethylating agent.

**Figure 2:**
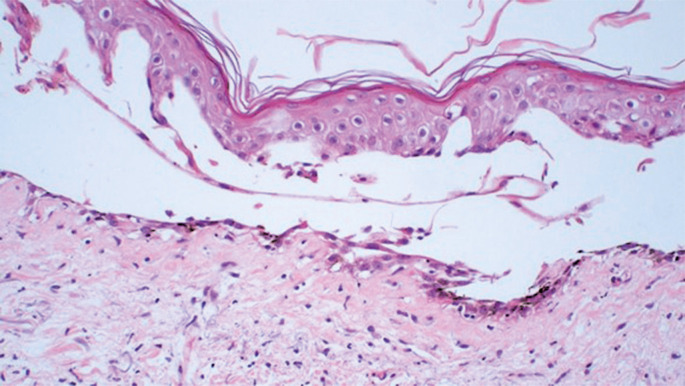
Skin biopsy showing subepidermal cleavage with re-epithelization and superficial and perivascular lymphohistiocytic infiltrate with sparse eosinophils

Histopathological examination of the skin lesion showed subepidermal cleavage with re-epithelization, and superficial and perivascular lymphohistiocytic infiltration with sparse eosinophils (Figure 2).

Bullous pemphigoid was ruled out by negative indirect immunofluorescence, absence of specific fluorescence on direct immunofluorescence, and the negative salt-split skin technique.

After medical board meetings, it was decided that further cycles of azacitidine should be administered intravenously rather than subcutaneously to decrease the risk of recurrence of cutaneous illness by eliminating the probable local factors involved.

During follow-up, after seven cycles of azacitidine, the patient underwent an erythroid response with red blood cell transfusion independence and without recurrence of the skin lesions since the end of the second cycle, with no need for topical or systemic medications to control lesions.

This study was approved by the Research Ethics Committee of *Hospital das Clínicas, Faculdade de Medicina, Universidade de São Paulo* (CAAE: 73843623. 4.0000.0068; #6.572.244).

## DISCUSSION

Bullous pemphigoid is an autoimmune blistering disease characterized by autoantibodies that recognize antigens in the basement membrane zone (BP180 and BP230).^5^ The diagnosis of this condition relies on clinical features, histological examination of skin biopsies, direct and indirect immunofluorescence investigations, and enzyme-linked immunoassay analysis.^5^

However, some entities presenting with multiple tense bullae in the absence of autoantibodies detectable by direct and indirect immunofluorescence studies or enzyme-linked immunoassay analysis may mimic bullous pemphigoids. Previous reports have shown the occurrence of bullous pemphigoid-like skin lesions associated with medications such as bendamustine,^6^ PD-1 inhibitors^7,8^and anticoagulants.^9,10^

To the best of our knowledge, no previous occurrence of bullous pemphigoid-like localized toxic reactions attributed to subcutaneous azacitidine application has been reported, although other forms of non-bullous cutaneous lesions related to azacitidine have been described.^4-6^

In our patient, the histopathological and immunofluorescence findings ruled out the diagnosis of bullous pemphigoid and suggested the diagnosis of a bullous pemphigoid-like localized toxic reaction. The strict anatomical and temporal correlation of the appearance of the lesions following administration of subcutaneous azacitidine; the absence of a prior history of trauma-induced bullae, especially in previous injections and venipunctures with metal needles; and the improvement of the skin alterations a few days after discontinuation of the medication suggest a drug-induced etiology, although a possible contributing traumatic factor due to subcutaneous needle insertion might also be present.

## CONCLUSION

It is proposed in this report that subcutaneous azacitidine administration has been shown to be a rare cause of bullous pemphigoid-like localized toxic reaction. We also argue that changing the route of azacitidine administration from subcutaneous to intravenous could potentially decrease the occurrence of this reaction.

Finally, a skin biopsy should be considered when a patient using subcutaneous medication presents with blistering lesions at the site of injection to confirm the diagnosis and plan correct measures to treat and prevent the recurrence of injuries.

## References

[1] Fenaux P, Mufti GJ, Hellstrom-Lindberg E, Santini V, Finelli C, Giagounidis A, Schoch R, Gattermann N, Sanz G, List A, Gore SD, Seymour JF, Bennett JM, Byrd J, Backstrom J, Zimmerman L, McKenzie D, Beach C, Silverman LR, International Vidaza High-Risk MDS Survival Study Group (2009). Efficacy of azacitidine compared with that of conventional care regimens in the treatment of higher-risk myelodysplastic syndromes: a randomised, open-label, phase III study. Lancet Oncol.

[2] Goldsmith SM, Sherertz EF, Powell BL, Hurd DD (1991). Cutaneous reactions to azacitidine. Arch Dermatol.

[3] Shimoda-Komatsu Y, Mizukawa Y, Takayama N, Ohyama M (2020). Cutaneous adverse events induced by azacitidine in myelodysplastic syndrome patients: Case reports and a lesson from published work review. J Dermatol.

[4] Waghmare P, Patra S, Thirunavukkarasu B, Bairwa S (2022). Azacytidine-induced Sweet's syndrome. BMJ Case Rep.

[5] Atteh G, Cole EF, Perricone AJ, Feldman RJ (2021). Bullous eczema presenting as bullous pemphigoid-like eruption: a case series. JAAD Case Rep.

[6] Civettini I, Bolis SA (2023). Bullous pemphigoid-like rash induced by bendamustine. Br J Haematol.

[7] Anastasopoulou A, Papaxoinis G, Diamantopoulos P, Christofidou E, Benopoulou O, Stratigos A (2018). Bullous Pemphigoid-like Skin Lesions and Overt Eosinophilia in a Patient With Melanoma Treated With Nivolumab: Case Report and Review of the Literature. J Immunother.

[8] Lomax AJ, Ge L, Anand S, McNeil C, Lowe P (2016). Bullous pemphigoid-like reaction in a patient with metastatic melanoma receiving pembrolizumab and previously treated with ipilimumab. Australas J Dermatol.

[9] Ferreira C, Oliveira A, Furtado A, Rocha N, Ribeiro JA (2018). Bullous Pemphigoid-like Skin Eruption during Treatment with Rivaroxaban: a Clinical Case Study. Eur J Case Rep Intern Med.

[10] Dyson SW, Lin C, Jaworsky C (2004). Enoxaparin sodium-induced bullous pemphigoid-like eruption: a report of 2 cases. J Am Acad Dermatol.

